# Comparison of the new Mycofast Revolution assay with a molecular assay for the detection of genital mycoplasmas from clinical specimens

**DOI:** 10.1186/1471-2334-13-453

**Published:** 2013-09-30

**Authors:** Mathys J Redelinghuys, Marthie M Ehlers, Andries W Dreyer, Hennie A Lombaard, Marleen M Kock

**Affiliations:** 1 Department of Medical Microbiology, University of Pretoria, Pretoria, South Africa; 2 Department of Medical Microbiology, Tshwane Academic Division, National Health Laboratory Service, Pretoria, South Africa; 3 Department of Obstetrics and Gynaecology, University of Pretoria, Pretoria, South Africa

**Keywords:** *Mycoplasma hominis*, *Ureaplasma* spp, Mycofast, Antimicrobial susceptibilities, Multiplex PCR assay

## Abstract

**Background:**

Genital mycoplasmas are opportunistic bacteria that are associated with undesirable gynaecologic and reproductive events. Mycoplasmas are fastidious bacteria with increasing resistance to routine antimicrobials and often fail to grow on conventional culture methods. The commercial Mycofast Revolution assay permits the phenotypic detection and identification of genital mycoplasmas. Antimicrobial susceptibility testing against five antimicrobial agents with MICs corresponding to the CLSI guidelines can also be performed. This study aimed to compare the new commercially available Mycofast Revolution assay with a multiplex PCR assay.

**Methods:**

Self-collected swabs were obtained from pregnant women attending the antenatal clinic of a tertiary academic hospital in Pretoria, South Africa from October 2012 to November 2012. These swabs were used to seed UMMt and modified Amies transport media. The seeded UMMt transported medium was used to inoculate the Mycofast Revolution assay for the identification, enumeration and antimicrobial susceptibility testing of genital mycoplasmas. Following DNA extraction from the modified Amies transport medium, specimens were subjected to a multiplex PCR assay for the detection of genital mycoplasmas.

**Results:**

The Mycofast Revolution kit had a sensitivity and specificity of 77.3% (95% CI: 62.15% to 88.51%) and 80% (95% CI: 28.81% to 96.70%), respectively, against the PCR assay. The positive and negative predictive values were 97.1% (95% CI: 85.03% to 99.52%) and 28.6% (95% CI: 8.57% to 58.08%). Genital mycoplasmas were detected in 71.4% (35/49) of samples with the Mycofast Revolution assay with 49% (24/49) being *Ureaplasma* spp. and 22.4% (11/49) mixed strains. The multiplex PCR assay had a positivity rate of 89.8% (44/49) for genital mycoplasmas; mixed strains were present in 51% (25/49) of samples, *Ureaplasma* spp. in 16.3% (8/49) and *M. hominis* in 22.4% (11/49) of samples.

**Conclusions:**

There was a fair agreement (κ = 0.319) between the Mycofast Revolution assay and the mPCR assay. With the high prevalence rates of genital mycoplasmas, fast and efficient diagnostic methods are imperative to treat infections and minimise complications. The Mycofast Revolution assay is simple to use, has a short turn-around time and interpretation of results are straightforward. This assay circumvents common problems experienced with conventional culture and molecular methods in diagnostic laboratories where skilled personnel are limited and can be used as an alternative diagnostic assay.

## Background

Genital mycoplasmas, including *Mycoplasma genitalium, M. hominis* and *Ureaplasma* spp. are potentially pathogenic bacteria that frequently colonise the genitourinary system of sexually active individuals [1]. Infections by these bacteria can lead to genital infections as well as undesirable sequelae during pregnancy [2,3]. The challenge of conventional methods to diagnose mycoplasmas forces researchers to investigate more sensitive, reliable and rapid alternatives. Susceptibility testing becomes prominent in the background of widespread antimicrobial resistance and topographical variation and must be incorporated in these testing systems.

Bacterial resistance to routine antimicrobial agents is a growing and worldwide problem. The lack of a rigid cell wall renders genital mycoplasmas innately resistant to antimicrobial agents, such as β-lactam antibiotics and vancomycin [4]. General treatment options include agents like tetracyclines and fluoroquinolones [5]. Fluoroquinolone antimicrobial agents can be used to treat genital mycoplasma infections caused by strains that are resistant to agents, such as the tetracycline agent doxycycline [6]. Agents that are frequently used include ofloxacin, ciprofloxacin, levofloxacin, gemifloxacin and moxifloxacin [7]. Moxifloxacin is a more recent quinolone, which has the highest *in vitro* activity against genital mycoplasmas [7]. These agents interact with the DNA gyrase and topoisomerase IV of bacteria [8]. Accordingly, fluoroquinolone resistance is associated with mutations in the *gyr*A and *gyr*B genes and the *par*C and *par*E genes [9]. Tetracyclines and fluoroquinolones are the drugs of choice, yet these agents are contraindicated in pregnancy [10,11]. During pregnancy, macrolides like erythromycin are often used [1,11].

Strains of *M. hominis* have natural resistance to C14 and C15 macrolides (e.g. clarithromycin, erythromycin, azithromycin and roxithromycin), while *Ureaplasma* spp. are resistant to lincosamides like clindamycin [12,13]. Resistance of *Ureaplasma* spp. to macrolides is widely reported and is associated with mutations in the 23S rRNA gene [14,15]. Tetracycline resistance is found in no less than 10% of *Ureaplasma* strains and approximately 40% of these resistant strains demonstrate cross-resistance to erythromycin [16]. Increased resistance to tetracyclines in *Ureaplasma* spp. and *M. hominis* is associated with the presence of the moveable *tet*(M) genetic element, the solitary tetracycline resistance mechanism, which renders ribosomes resistant to this agent [17,18].

Phenotypic and genotypic methods for the identification of mycoplasmas are available. Culture is still regarded as the gold standard for the detection of recoverable bacteria like *M. hominis* and *Ureaplasma* spp.; however, a low sensitivity when compared to polymerase chain reaction (PCR) assays has been reported [19,20]. Culture is labour intensive and time consuming as it requires the use of an enrichment broth for up to seven days, followed by sub-culturing on solid media. Analytical sensitivities in the range of 60% are only obtained in skilled laboratories and identification is restricted to the genus level. The development of commercially available diagnostic assays, which are based on liquid broth cultures provide easy to use and faster alternatives to conventional culture methods for the detection of genital mycoplasmas [21]. The difficulty of laboratory culture methods to isolate *M. genitalium* complicates antimicrobial susceptibility testing [22]. There is currently no approved commercially available diagnostic assay for the detection and antimicrobial resistance testing of *M. genitalium*; detection is mainly done by nucleic acid amplification tests (NAATs) [23].

The new commercially available Mycofast Revolution (ELiTech Diagnostic, France) assay is a CE approved assay (European Conformity; A mandatory European marking for certain product groups to indicate conformity with the essential health and safety requirements set out in European Directives). This assay provides easy identification and enumeration of *M. hominis* and/or *Ureaplasma* spp. within 24 h to 48 h [24]. The Mycofast Revolution assay is a liquid method based on the ability of *Ureaplasma* spp. and *Mycoplasma hominis* to metabolize urea and arginine, respectively and consists of 20 wells that are pre-coated with a dehydrated culture medium (foal serum, yeast extract, cysteine, arginine, urea, phenol red and antibiotics) and contains a single broth with antimicrobials for transport and preservation of genital mycoplasmas (UMMt) (ELiTech Diagnostic, France). The Mycofast Revolution assay includes an additional screening tray, which can be used prior to inoculation to differentiate between positive and negative specimens and is much more cost-effective. The screening tray and test trays allow the detection of genital mycoplasmas at concentrations ≤10^3^ colour change units per millilitre (ccu/ml) and >10^3^ ccu/ml, respectively.

Other commercially available diagnostic assays that are similar with regards to genital mycoplasma identification, antimicrobial susceptibility testing, turn-around time and ease of use include the Mycoplasma Duo kit (Sanofi Diagnostics Pasteur, France), the Mycoview (Ivagen) and MycoIST2 (BioMérieux) test kits [14,25]. The advantage of the Mycofast Revolution assay is that antimicrobial susceptibility testing is performed against different antimicrobial agents with specific minimum inhibitory concentrations (MICs) as defined by the 2011 Clinical and Laboratory Standards Institute (CLSI) guidelines. Antimicrobial susceptibility testing is performed against five antimicrobial agents that include levofloxacin, moxifloxacin, erythromycin, clindamycin and tetracycline [24].

Molecular methods, such as PCR assays are reported to be more sensitive for diagnostic purposes than culture [26]. Waites *et al.*[26] indicated that PCR-positive, culture-negative specimens are likely to represent true positives due to the much higher sensitivity. Genotypic methods also allow for speciation, which is a limitation of culture [26]. Other advantages include rapid detection as well as that these assays do not rely on the viability of the bacterium for detection [27]. Furthermore, when using a multiplex PCR (mPCR) assay, the detection of more than one target in a single reaction is possible and this can simplify the workflow [28].

The new commercially available Mycofast Revolution assay may have the potential to be used as a simplified and cost effective method to diagnose genital mycoplasmas. The purpose of the study was to compare the Mycofast Revolution assay with an mPCR assay for the detection of genital mycoplasmas from clinically collected vaginal specimens.

## Methods

The study was conducted at the Department of Medical Microbiology, University of Pretoria from October 2012 to November 2012. Ethical approval was obtained from the Student Research Ethics Committee of the University of Pretoria prior to commencement of the study (Approved protocol number: S6/2012). The study population included pregnant women attending the antenatal clinic at a tertiary academic hospital in Pretoria, Gauteng, South Africa. The inclusion criteria of participants in this study were pregnant women attending the antenatal clinic who were older than 18 years and who gave written informed consent. All non-pregnant women, pregnant women younger than 18 years or pregnant women who did not give written informed consent were excluded from this study.

Two self-collected vaginal swabs (Copan Diagnostics, Inc, Italy) were obtained from fifty pregnant women. The order in which the swabs were obtained was randomised between patients. After collection, a dry Rayon swab was used to seed 3 ml transport (UMMt) medium of the Mycofast Revolution assay; the second swab (a flocked nylon swab) was inoculated into 1 ml of modified Amies transport medium (Copan Diagnostics, Inc, Italy) and used for PCR analysis. Inoculated media and reagents used were stored at 2°C to 8°C, whereas consumables were stored at room temperature (±25°C). The inoculated modified Amies transport medium was stored at −20°C until DNA extraction was performed (within ±2 weeks of specimen collection). Extracted DNA was stored at −20°C until PCR analysis (done within ±1 week after DNA extraction).

The swabs and the transport media were processed according to the manufacturer’s instructions. Briefly, 100 μl of seeded UMMt medium was added to the *M. hominis* (MH) and *U. urealyticum* (UU) wells of the Mycofast Screening Revolution tray with an additional 50 μl of MH supplement (S.Mh) added to the MH well. The wells were covered with two drops of sterile mineral oil and the tray was incubated (Vacutec, South Africa) at 37°C ± 1°C for 24 h. After incubation, the wells were observed for any colour changes. Orange or red colour changes indicated the presence of *M. hominis* and/or *Ureaplasma* spp., whereas yellow wells marked the absence of mycoplasmas. In the case of a positive screening test, the excess UMMt medium that was stored at 2°C to 8°C was used to inoculate the Complement Mycofast Revolution tray. Wells 1 to 20 were filled with 100 μl of seeded UMMt medium, wells 6 to 7 filled with an additional 50 μl of S.Mh and all the wells were covered with two drops of mineral oil. The tray was incubated (Vacutec, South Africa) at 37°C ± 1°C for 24 h (maximum 48 h in all cases) and after incubation observed for colour changes similar to that of the screening tray. *Mycoplasma hominis* (MH) identification wells contained erythromycin to inhibit the growth of *Ureaplasma* spp., while the UU wells contained lincomycin to inhibit the growth of *M. hominis*.

The specific breakpoints (in μg/mL) indicating susceptibility (S) or resistance (R) for *Ureaplasma* spp. are as follow [24]: levofloxacin S ≤ 2, R ≥ 4; moxifloxacin S ≤ 2; erythromycin S ≤ 8, R ≥ 16; tetracycline S ≤ 1, R ≥ 2. The breakpoints for *M. hominis* are as follow: levofloxacin S ≤ 1, R ≥ 2; moxifloxacin S ≤ 0.25; clindamycin S ≤ 0.25, R ≥ 0.5; tetracycline S ≤ 4, R ≥ 8. Strains were regarded as resistant when growth was inhibited by the higher critical concentration of the antimicrobial agent, but not the lower critical concentration or when growth was not inhibited by either the higher or lower critical concentrations of the antimicrobial agents.

Specimens were subjected to a human β-globin PCR assay to serve as an internal control and monitor possible PCR inhibitors. The mPCR assay used was done according to Stellrecht *et al*. [29] but in multiplex format with the following modifications: 40 PCR cycles and primers at final concentrations of 0.2 μM with the Qiagen multiplex PCR kit (Qiagen, Germany). This method was previously compared to the gold standard (culture on A7 agar) for genital mycoplasma identification and showed good sensitivity, specificity and positive and negative predictive values (87%, 96%, 94% and 93%, respectively) for the detection of genital mycoplasmas [29]. The mPCR assay was conducted with primers targeting genes specific for *M. genitalium, M. hominis*, *U. parvum* and *U. urealyticum*[29]. Oligonucleotide primers were synthesised by Inqaba Biotechnical Industries, South Africa. The mPCR assay was validated with AmpliRun *Mycoplasma genitalium* DNA control (Vircell SL, Spain), a positive *M. hominis* specimen isolated with A2 agar and reference strains ATCC 27813 (*U. parvum*) and ATCC 27619 (*U. urealyticum*).

Statistical analysis was performed using the PCR assay as the gold standard to calculate the sensitivity, specificity, positive predictive value and negative predictive value of the Mycofast Revolution assay. The positivity rates of both assays were determined and the agreement between the two methods was determined by the kappa (κ) statistic. The κ value, a measure of test reliability, was interpreted as follows: < 0.2, poor; 0.21 to 0.4, fair; 0.41 to 0.6, moderate; 0.61 to 0.8, good; ≥ 0.81, excellent [30].

## Results

A total of 49 samples were included in this study. Contamination was observed in one specimen (2%) that was excluded from the analysis. The number of specimens that tested positive and negative with the Mycofast Revolution (phenotypic) and the mPCR (genotypic) assays as well as the breakdown according to species are displayed in Table 1.

**Table 1:** **Results of *M. hominis *and *Ureaplasma *spp. after the Mycofast Revolution and mPCR assay analyses (n = 49)**

**Assay**	***M. hominis *(MH)**^**1 **^**No (%)**	***Ureaplasma *spp. (UU + UP)**^**1 **^**No (%)**	**Mixed isolation: MH + (UU + UP) No (%)**	**Negatives No (%)**	**Total**
**Mycofast Revolution**	0	24 (49)	11 (22.4)	14 (28.6)	49
**PCR**	11 (22.4)	8 (16.3)	25 (51)	5 (10.2)	49

^1^Where MH is *M. hominis*, UP is *U. parvum* and UU is *U. urealyticum*.

Genital mycoplasmas were detected in 71.4% (35/49) of samples with the Mycofast Revolution assay. Forty-nine percent (24/49) of cultures were positive for *Ureaplasma* spp., while none of the cultures were positive for only *M. hominis*. Mixed strains (*M. hominis* and *Ureaplasma* spp.) were present in 22.4% (11/49) of cultures. Mixed strains were determined when the identification wells of both *M. hominis* and *Ureaplasma* spp. gave positive results. Mycoplasmas were not detected in 28.6% (14/49) of specimens. One sample was positive with the Mycofast Revolution assay but negative with the mPCR assay.

*Ureaplasma* spp. were resistant to levofloxacin and moxifloxacin in 42% (10/24) and 4% (1/24) of cases, respectively (Table 2). *Ureaplasma* spp. had susceptibilities of 25% (6/24) and 21% (5/24) to erythromycin and tetracycline, respectively. The resistance patterns for mixed isolates were similar to those of *Ureaplasma* spp., except for erythromycin and tetracycline to which 100% (11/11) of the isolates were resistant.

**Table 2:** **The distribution (%) of *Ureaplasma *spp. and *M. hominis *at different breakpoints of antimicrobial agents (n = 49)**

	**Levofloxacin**			**Moxifloxacin**		**Erythromycin**		**Clindamycin**		**Tetracycline**			
	**1**^**1**^	**2**	**4**	**0.25**	**2**	**8**	**16**	**0.25**	**0.5**	**1**	**2**	**4**	**8**
***Ureaplasma *species (n = 24)**	50	29	13	88	4	71	4	0	100	17	25	4	33
***Ureaplasma *species and *M. hominis *(n = 11)**	73	18	9	82	9	9	91	0	100	9	18	9	64

^1^The breakpoints in μg/mL according to the CLSI guidelines^24^.

The mPCR assay detected genital mycoplasmas in 89.8% (44/49) of specimens. *Ureaplasma* spp. were detected in 16.3% (8/49), while *M. hominis* was detected in 22.4% (11/49) of specimens. Fifty-one percent (25/49) of specimens were positive for both *Ureaplasma* spp. and *M. hominis*. The mPCR assay results showed only 10.2% (5/49) of specimens to be negative.

Statistical analysis, when considering the mPCR assay as the gold standard, showed a sensitivity and specificity of 77.3% (95% CI: 62.15% to 88.51%) and 80% (95% CI: 28.81% to 96.70%), respectively for the Mycofast Revolution assay to detect genital mycoplasmas. The positive and negative predictive values were 97.1% (95% CI: 85.03% to 99.52%) and 28.6% (95% CI: 8.57% to 58.08%), respectively. The kappa statistic was 0.319.

## Discussion

This study is the first to compare the Mycofast Revolution commercial assay against an mPCR assay for the detection of genital mycoplasmas from clinical specimens in South Africa. There was a fair agreement (κ = 0.319) between the results of the phenotypic and genotypic methods. The Mycofast Revolution assay showed a high sensitivity and specificity, of 77% and 80% respectively, considering it only detects viable bacteria. However, this contributed to a low negative predictive value (28.6%) when the mPCR assay was considered the gold standard.

The positivity rates reported in this study are high (71% for the Mycofast Revolution and 91.8% for the mPCR assays). A study by Bayraktar *et al*. [31] in pregnant women, including symptomatic and asymptomatic control patients, reported a prevalence of 29% for genital mycoplasmas. A Greek study (2009) reported a prevalence of 37% in outpatient women with clinical vaginitis [32]. Both of these studies identified genital mycoplasmas with the commercially available Mycoplasma IST-2 kit. Govender *et al*. screened low-risk antenatal patients in South Africa at their first antenatal visit (16 to 23 weeks’ gestation) for mycoplasmas at two different time frames (2003 and 2005) [3]. This research group used an mPCR assay and documented prevalence rates of genital mycoplasmas of almost 80% and around 40% in 2003 and 2005, respectively [3]. Nonetheless, the type of assay may have an effect on the accurate detection of genital mycoplasmas, depending on the growth factors and antimicrobial agents included in the media of the commercial assay.

A higher detection rate was observed for *Ureaplasma* spp. (detected alone in 24% of specimens) compared to *M. hominis* (never detected alone) with the Mycofast Revolution assay*.* The opposite was true for the mPCR assay with *M. hominis* being detected more frequently (detected alone in 22.4% of specimens, 6.1% more than the single detection of *Ureaplasma* spp.). The reason for the higher detection rate by the mPCR assay could be ascribed to specimens containing a low concentration of bacteria that were not detected after 48 h with the Mycofast Revolution assay*.* In such cases, the presence of genital mycoplasmas may possibly be colonisation instead of infection as the Mycofast Revolution assay is designed to detect whether the pathological threshold was reached.

A limitation of the Mycofast Revolution assay is that a low concentration of *M. hominis* may result in random wells to turn positive. Nonetheless, a specimen would only be regarded as positive if the identification wells are positive and the pathological thresholds are reached. The Mycofast Revolution assay does not distinguish between the species, *U. parvum* and *U. urealyticum* and analysis with additional molecular methods is needed for speciation. The specimen which tested positive with the Mycofast Revolution assay but negative with the PCR assay was neither re-extracted nor repeated with a PCR assay.

Although PCR assays have the advantage of being sensitive, it remains costly and is dependent on skilled personnel. The inoculation and handling of the Mycofast Revolution assay do not require skilled personnel and the results are easy to interpret. In addition, the Mycofast Revolution assay allows quantitation of the number of mycoplasmas present and gives an indication of colonisation or infection. Despite the lower observed sensitivity of the Mycofast Revolution assay compared to the mPCR assay, the main advantage of the Mycofast Revolution assay is that it tests the activity of a variety of new antimicrobial agents against genital mycoplasmas with updated MICs as defined by the 2011 CLSI guidelines [24]. This may reduce the cost of antimicrobial surveillance and renders the Mycofast Revolution assay of clinical importance in the era of increasing antimicrobial resistance. The Mycofast Revolution assay may be an acceptable assay to use in routine diagnostic laboratories in South Africa where resources are limited. It may be used as an alternative in laboratories where insensitive conventional culture methods are used.

The difference in the findings between the two assays can be ascribed to numerous factors, including different bacterial loads on the different swabs, the viability of bacteria and the difference in analytical sensitivities of the two assays. A limitation of the study was that an additional molecular assay was not used to resolve the discrepancies between the two assays.

## Conclusions

The Mycofast Revolution assay could be considered as a cost-effective alternative to conventional culture methods for the rapid detection of genital mycoplasmas. The assay may allow laboratory personnel to provide the clinician with a result in a short period (± 48 hours) of time together with antimicrobial susceptibility data. Antimicrobial susceptibility patterns are vital as successful treatment will depend on the early administration of effective antimicrobial agents. In pregnant women it is particularly important to reduce these infections to prevent adverse pregnancy outcomes.

## Competing interests

None to declare. The authors would like to thank Separation Scientific for supplying the Mycofast Revolution kits used in this study. The authors would also like to thank the University of Pretoria, the Medical Research Council (South Africa) and the National Health Laboratory Service (NHLS) for financial assistance received.

## Authors’ contributions

MJR was involved in concept design, laboratory work as well as writing of the manuscript. MMK, MME and AWD were involved in concept design of the study as well as critical review of the manuscript. HL was involved in concept design of the study as well as overseeing the logistics of sample collection. All authors read and approved the final manuscript.

## Pre-publication history

The pre-publication history for this paper can be accessed here:

http://www.biomedcentral.com/1471-2334/13/453/prepub
